# Identifying genomic regions for fine-mapping using genome scan meta-analysis (GSMA) to identify the minimum regions of maximum significance (MRMS) across populations

**DOI:** 10.1186/1471-2156-6-S1-S42

**Published:** 2005-12-30

**Authors:** Margaret E Cooper, Toby H Goldstein, Brion S Maher, Mary L Marazita

**Affiliations:** 1 Center for Craniofacial and Dental Genetics, School of Dental Medicine, University of Pittsburgh, Pittsburgh PA 15219, USA

## Abstract

In order to detect linkage of the simulated complex disease Kofendrerd Personality Disorder across studies from multiple populations, we performed a genome scan meta-analysis (GSMA). Using the 7-cM microsatellite map, nonparametric multipoint linkage analyses were performed separately on each of the four simulated populations independently to determine *p*-values. The genome of each population was divided into 20-cM bin regions, and each bin was rank-ordered based on the most significant linkage *p*-value for that population in that region. The bin ranks were then averaged across all four studies to determine the most significant 20-cM regions over all studies. Statistical significance of the averaged bin ranks was determined from a normal distribution of randomly assigned rank averages. To narrow the region of interest for fine-mapping, the meta-analysis was repeated two additional times, with each of the 20-cM bins offset by 7 cM and 13 cM, respectively, creating regions of overlap with the original method. The 6–7 cM shared regions, where the highest averaged 20-cM bins from each of the three offsets overlap, designated the minimum region of maximum significance (MRMS). Application of the GSMA-MRMS method revealed genome wide significance (*p*-values refer to the average rank assigned to the bin) at regions including or adjacent to all of the simulated disease loci: chromosome 1 (*p *< 0.0001 for 160–167 cM, including D1), chromosome 3 (*p*-value < 0.0000001 for 287–294 cM, including D2), chromosome 5 (*p*-value < 0.001 for 0–7 cM, including D3), and chromosome 9 (*p*-value < 0.05 for 7–14 cM, the region adjacent to D4). This GSMA analysis approach demonstrates the power of linkage meta-analysis to detect multiple genes simultaneously for a complex disorder. The MRMS method enhances this powerful tool to focus on more localized regions of linkage.

## Background

After a genome scan, fine-mapping of the most promising regions proceeds. Identification of the regions must be as accurate as possible to minimize time and expense. In complex diseases, there are often many research groups working independently but cooperatively. A meta-analysis of the genome scans from diverse research groups can reveal the appropriate areas for fine-mapping. We proposed to use the results from the individual genome scans of the Genetic Analysis Workshop simulated populations in a meta-analysis to assess the optimal chromosomal region(s) to target for second stage fine-mapping. The genome scan meta-analysis (GSMA) [1,2] method is a nonparametric rank ordering method that can combine genome-scan methods across studies with different markers, and/or different statistical tests, and is robust to study design and ascertainment differences. In simulation studies, the GSMA detected linkage with power comparable to or greater than that obtained by performing a combined linkage analysis of all the data [2]. An extension of the GSMA method to determine the minimum regions of maximum significance (MRMS) is used for revealing areas for fine-mapping in complex diseases [3].

## Methods

### GSMA method

Linkage between traits and markers was assessed via nonparametric multipoint linkage methods. For the multigenerational New York families, we used the descent graph approach, utilizing computer program SIMWALK V2.89 [4], and MEGA2 V2.5.R4 utility program [5,6]. For the nuclear families of the other 3 populations, we used MERLIN 0.10.1 [7]. Family data from all populations from replicate 1 was used and the affection trait investigated was the overall affection status of Kofendrerd Personality Disorder.

For the GSMA procedure, the genome was divided into 20-cM regions, with bin width selected such that there were at least 2 bins on each chromosome and at least one marker in each bin. For each of the 4 scans, bins were assigned a rank (*R*, with values 1–144) according to the most significant *p*-value of any markers within that bin. Any ties were assigned equal ranks on the basis of the mean of the sequential ranks for those bins. Higher values of *R *represented the most significant *p*-values.

For each bin, the ranks were summed and averaged over all four populations. Each population carried the same weight.

A weighting scheme was considered because of the differing sample size of the populations and differing numbers of affecteds in each family due to the ascertainment criteria. The weighting scheme factor [2] depended on the square root of the number of affecteds genotyped in each study (N) divided by the mean of affecteds genotyped for all 4 studies  The weights calculated were close to 1.0, between 0.95 and 1.03, and therefore weighting was not considered necessary.

Because no weighting scheme was used, statistical significance of the average rank was determined by the normally distributed probability function derived by assuming that each of the independent possible average ranks were randomly assigned [1].

### Extension of GSMA to find MRMS

To narrow the regions of possible findings, we utilized an extension of the GSMA procedure. We repeated the GSMA procedure twice, assigning different bins to the map: shortening the length of the first bin to 7 cM, then to 13 cM, but kept all subsequent bins to a length of 20 cM. Thus we were able to determine the 6- to 7-cM region overlap that was the minimum region of maximum significance (MRMS) [3]. Given that the scans averaged 7.5 cM between markers, the 6 to 7 cM was the limit of resolution for this meta-analysis.

Analysis proceeded without knowledge of the simulated disease loci.

## Results

**Figure 1:**
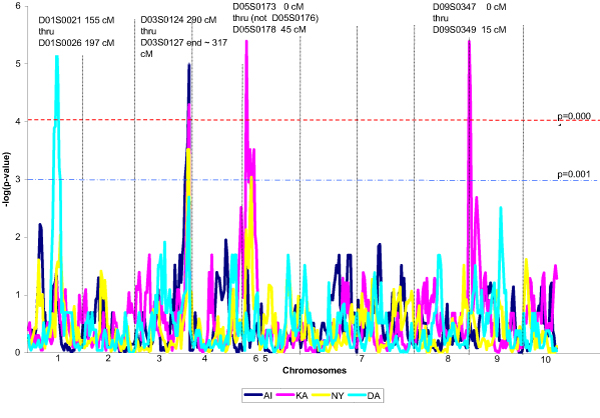
**Raw nonparametric linkage multipoint results**. The red dashed line represents the 0.05 genome-wide significance level adjusted for multiple tests (Bonferroni adjustment). The other blue line indicates the unadjusted *p*-value of 0.001.

Multipoint results in the four populations (Figure 1) indicated 19 markers on 4 chromosomes with raw *p*-values less than 0.001. Many more markers had raw *p*-values < 0.05. There were 7 markers on 4 chromosomes that met the Bonferroni adjusted significance requirement, yet 6 of these markers were significant in only one population (D01S0022, D010023, D01S0024 in Danacaa and D05S0172, D09S0347, D09S0348 in Karangar) and 1 marker (D03S0127) was significant in 2 populations (Aipotu and Karangar).

**Figure 2:**
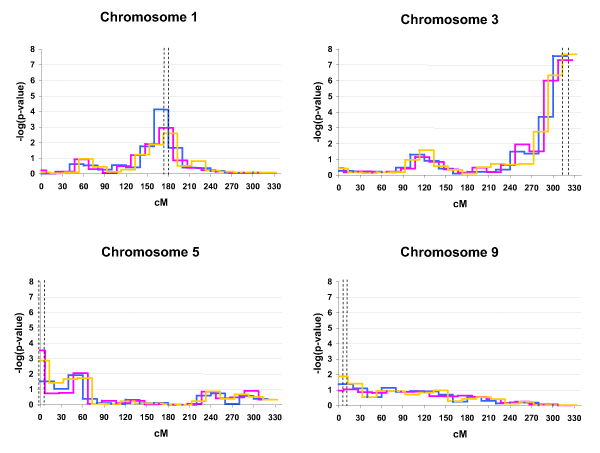
**Graphic representation of the minimum regions of maximum significance (MRMS) for those chromosomes with GSMA-MRMS values < 0.01**. The dashed vertical lines indicate the 6–7 cM MRMS region for chromosomes 1, 3, 5, and 9 and therefore the regions targeted for SNP packet purchasing for fine-mapping.

The bin-shifting procedure and the MRMS method (Figure 2) identified 4 regions with genome-wide significance for second stage fine-mapping: chromosome 1: 173 to 180, chromosome 3: 313 cM to the end, chromosome 5: 0 to 7 cM, and chromosome 9: 7–13 cM. Regions adjacent to the left of these 4 regions arguably could also be included in fine-mapping, money and resources permitting. Therefore, our proposed approach to combine data across diverse populations (GSMA plus MRMS) correctly identified the simulated disease regions on chromosome 1, 3, 5 and the adjacent region on chromosome 9.

## Discussion

The GSMA-MRMS procedure correctly identified the 3 disease regions on chromosomes 1, 3, and 5. The fourth disease region on chromosome 9 revealed by GSMA-MRMS was directly adjacent to the simulated disease region. We believe that the GSMA-MRMS method is superior to other methods that might be used to identify localized regions of linkage. Without the shifting of the bins (MRMS method), the GSMA alone would have indicated a 20-cM region on each of the chromosomes 1,3, 5, and 9, effectively tripling the cost and time of the fine-mapping procedure. Using just the Bonferroni-corrected *p*-values from the multipoint analysis, 3 regions varying from 14 to 33 cM would have been considered for fine-mapping on chromosomes 1, 3, and 5. Using *p*-values < 0.001 from the multipoint analysis, even larger regions varying from 24 to 44 cM would have been considered for fine-mapping on chromosomes 1, 3, and 5. The GSMA-MRMS enhanced method, in comparison to the alternative methods presented above, would be the most cost effective method for identifying regions for second stage fine-mapping.

## Conclusion

The GSMA method alone identified 20-cM regions while the GSMA method followed by the MRMS narrowed the regions to consider, leading to more efficient use of time, resources and funds for follow-up fine-mapping studies. With many investigators focusing on complex diseases with sometimes conflicting findings from study to study, and with the necessity to combine data across studies with potentially different study designs, the GSMA-MRMS methodology would expedite the discovery of a complex disease's genetic basis.

## Abbreviations

GSMA: Genome scan meta-analysis

MRMS: Minimum regions of maximum significance

## Authors' contributions

MEC completed the genetic analysis, identified the bins, and calculated the weights and the normal distribution of ranks used for the *p*-values. THG completed the simulations for the empiric *p*-values of the weighted data. BSM devised the methodology for the simulation of the weighted data. MLM created the method of bin shifting to narrow the regions of maximum significance to address the concerns of MEC, THG and BSM that the GSMA methods alone might lead to misleading results, depending on the placement of the bins.
